# Supplementary material for: Reflection confocal microscopy for quantitative assessment of airway surface layer dysregulation and pharmacological rescue in cystic fibrosis under near-physiological conditions
Source: Sci Rep. 2025 Dec 11;15:43659. doi: 10.1038/s41598-025-32061-3 (PMC12701019; doi:10.1038/s41598-025-32061-3)
Supplement: Supplementary file 1 — Supplementary Material 1 [file 41598_2025_32061_MOESM1_ESM.docx]

Supplemental figure 1


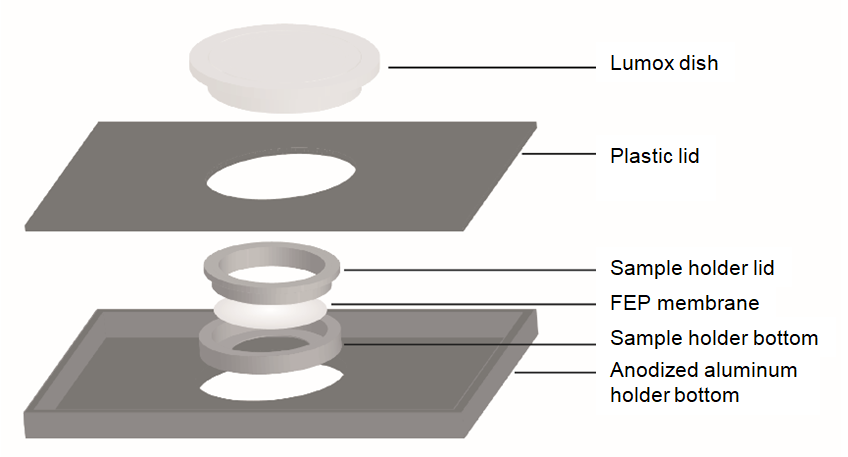


Schematic representation of the reflection microscopy sample holder set up. The Transwell is placed on the FEP membrane and the sample holder is closed. The sample holder is placed in the aluminum holder with the water reservoir and closed with the plastic lid and the gas-permeable bottom part of the lumox dish.
