# Supplementary material for: Reflection confocal microscopy for quantitative assessment of airway surface layer dysregulation and pharmacological rescue in cystic fibrosis under near-physiological conditions
Source: Sci Rep. 2025 Dec 11;15:43659. doi: 10.1038/s41598-025-32061-3 (PMC12701019; doi:10.1038/s41598-025-32061-3)
Supplement: Supplementary file 2 — Supplementary Material 2 [file 41598_2025_32061_MOESM2_ESM.docx]

Supplemental figure 2


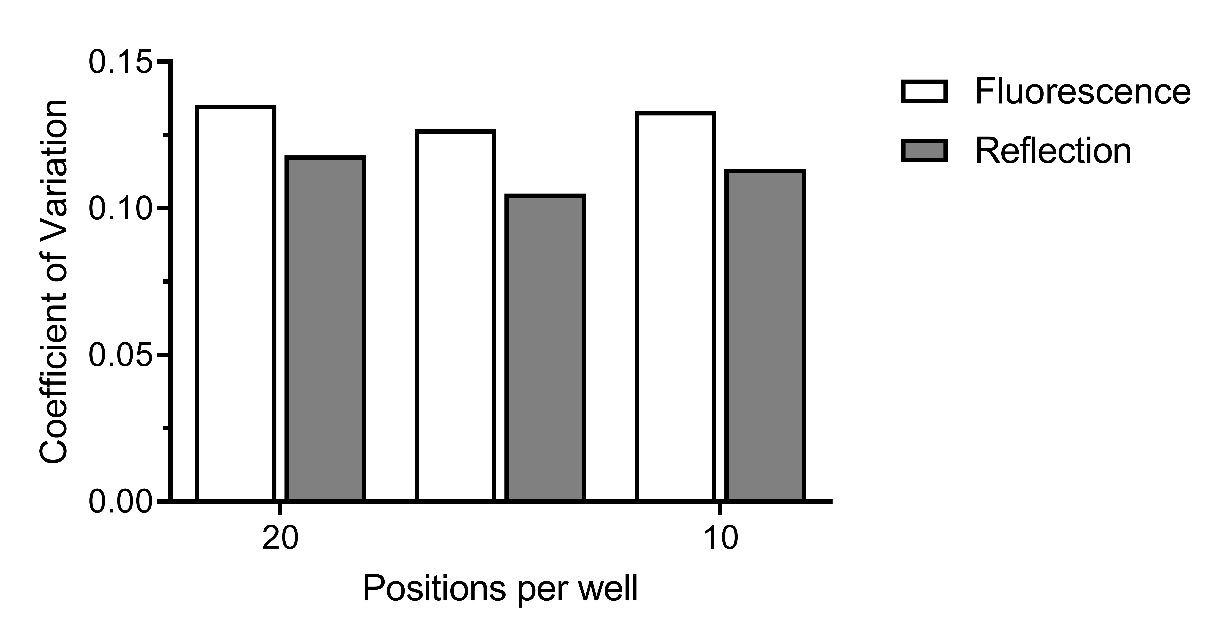


15

Reanalysis of subsampled datasets for 10, 15 and 20 positions per culture comparing both the fluorescence confocal microscopy and the new reflective technique show that the coefficients of variation exhibited minimal changes, supporting the conclusion that 15 images per culture sufficiently capture variability.
